# Case Report: melanoma and melanocytic nevus differentiation with reflectance confocal microscopy.

**DOI:** 10.12688/f1000research.6793.1

**Published:** 2015-07-15

**Authors:** Joanna Łudzik, Alexander M Witkowski, Giovanni Pellacani

**Affiliations:** 1 Department of Dermatology, University of Modena and Reggio Emilia, Modena, 41124, Italy; 2 Department of Biostatistics and Telemedicine, Jagiellonian University Medical College, Krakow, 31-530, Poland

**Keywords:** Reflectance confocal microscopy, Melanoma, Dysplasia, Nevi, Dermatology

## Abstract

Historically, melanoma has been typically diagnosed by naked-eye examination and confirmed with invasive biopsy. However, recently the use of reflectance confocal microscopy enables non-invasive bedside diagnosis of clinically equivocal lesions. We present a case in which reflectance confocal microscopy was used to evaluate two skin lesions in the same patient confirming the diagnosis of a melanoma and potentially avoiding invasive biopsy in the second benign melanocytic lesion.  Clinicians should be aware of the availability of new non-invasive technologies that can aid in early diagnosis of malignant skin tumors and potentially reduce the number of benign lesion excisions.

## Introduction

We report a case of a patient with multiple atypical melanocytic nevi evaluated with dermoscopy and reflectance confocal microscopy during a referral skin cancer control visit.

## Background

Skin tumor diagnosis can be difficult due to the variable clinical presentation of skin lesions. In order to correctly identify melanoma at its earliest stage, the use of dermoscopy has been shown to significantly increase the sensitivity and specificity of diagnosis when compared to traditional naked-eye examination ^1, 2^. In equivocal cases benign lesions may be excised when further cytological information is required to rule out malignancy. Recently reflectance confocal microscopy (RCM) use in clinical practice has been shown to further improve early melanoma diagnosis non-invasively by providing an *in-vivo* optical biopsy at histologic resolution down to a depth of 200 µm of skin tissue ^3– 7^. Moreover RCM has been shown to significantly reduce the number of unnecessary excisions in different settings ^8– 10^.

In this article we review the clinical, dermoscopic, and RCM presentation of two lesions in the same patient controlled with the gold standard of histopathology diagnosis.

## Case report

A 65 year old Caucasian female (Fitzpatrick skin type III) presented to the dermatology department at the University of Modena and Reggio Emilia (UNIMORE) with referral from a general practitioner for two skin lesions. Her past medical history and family history were negative for melanoma. No other significant medical history was noted. The patient reported having several invasive biopsies of dysplastic nevi in the past, the last reported biopsy in 2012. In 2013 the patient had her last naked-eye skin cancer screening by a private dermatologist with no worrisome skin lesions identified or recommended for biopsy.

## Clinical naked-eye examination findings

**Figure 1.**
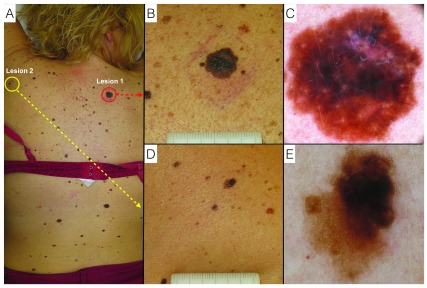
Melanoma and dysplastic nevus. **A**. Clinical overview of the patient. **B**. Lesion 1: Melanoma - naked-eye clinical close-up. **C**. Lesion 1: Melanoma - digital dermoscopy view. **D**. Lesion 2: Dysplastic nevus - naked-eye clinical close-up. **E**. Lesion 2: Dysplastic nevus - digital dermoscopy view.

The patient presented with a high numerosity of multiple irregularly shaped nevi located mainly on the back and lower legs. Lesion number 1, located on the upper right back, presented with ABCD (asymmetry, irregular borders, multiple colors, diameter >6mm) positive criteria and was of highest concern as it was the largest solitary macule on the back. Lesion number 2, located on the upper left shoulder, also presented with ABCD (asymmetry, irregular borders, multiple colors, diameter >6mm) positive criteria ( Figure 1A, B, D).

## Digital dermoscopy findings

Dermoscopy evaluation was performed with both a handheld dermatoscope and sequential digital dermoscopy (videodermoscopy). Lesion 1 presented with dermoscopic findings including: asymmetry, irregular reticular network with areas of eccentric hyperpigmentation, blue-white areas, and peppering representing early regression ( Figure 1C). Lesion 2 presented with dermoscopic findings including: asymmetry and eccentric hyperpigmented network ( Figure 1E).

## Reflectance confocal microscopy findings

After dermoscopic evaluation and storage of both lesions in the UNIMORE digital dermoscopy database system the patient was referred for further evaluation with reflectance confocal microscopy. RCM images were obtained with a reflectance confocal microscope (Vivascope1500; MAVIG GmBH, Munich, Germany) using a 830 nm laser at a maximum power of 20 mW. RCM images of 0.5 × 0.5 mm were acquired with a lateral resolution of 1 μm and an axial resolution of 3–5 μm and stitched into composite images that covered between 4 to 8 square mm mosaics (VivaCube; MAVIG GmBH, Munich, Germany). A minimum of three mosaics were obtained at different depths, corresponding to the stratum granulosum/spinosum, the dermo-epidermal junction, and the papillary dermis.

**Figure 2.**
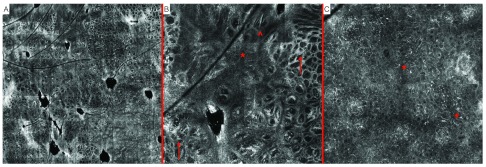
Melanoma. Reflectance confocal microscopy (RCM) imaging. **A**. Mosaic-map overview. **B**. Presence of non-specific pattern (*), atypical meshwork pattern (↑), aggregates of dendritic-type atypical melanocytes in bundles (^), location: dermo-epidermal junction. **C**. Disarrangement of the epidermis with an atypical honeycombed pattern and presence of a high numerosity of heterogeneously shaped pagetoid cells (*), location: epidermis.

Lesion number 1 presented with the following findings at the dermo-epidermal junction: predominant meshwork architecture composed of enlarged interpapillary spaces with junctional nests containing atypical melanocytes. Additionally, there were areas of loss of architectural structure and replacement by non-specific architecture with bundles of atypical dendritic-type melanocytes. The epidermis presented with complete disarrangement with an atypical honeycombed pattern and presence of a high numerosity of heterogeneously shaped pagetoid cells ( Figure 2). RCM examination was therefore confirming the diagnosis of melanoma, later confirmed by histopathology report.

**Figure 3.**
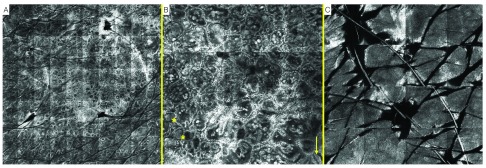
Dysplastic nevus. Reflectance confocal microscopy (RCM) imaging. **A**. Mosaic-map overview. **B**. Presence of ringed and clod (*) architecture, representing junctional lentiginous proliferation of melanocytes and dermal nests respectively, location dermo-epidermal junction. **C**. Regular honeycombed pattern with few inflammatory cells, location: epidermis.

Lesion number 2 presented with the following findings at the dermo-epidermal junction (DEJ): predominant ringed and clod architecture, representing junctional lentiginous proliferation of melanocytes and dermal nests respectively, with absence of atypical cells. Additionally at the DEJ there were few areas of meshwork architecture. The epidermis presented with a regular honeycombed pattern with few inflammatory cells ( Figure 3). RCM examination was therefore suggestive of a dysplasic nevus, later confirmed by histopathology report.

## Discussion

The purpose of our case-report was to present a typical scenario encountered by clinicians in daily practice where multiple lesions are referred for skin cancer examination. The methodology of full body dermoscopy evaluation to identify potentially high risk skin lesions and further evaluation with RCM imaging provides trained experts with cellular information about skin lesions non-invasively at the bedside. Ultimately this information can aid in early diagnosis of malignant skin tumors and moreover potentially reduce removal of benign lesions, saving patients from unnecessary scaring and healthcare costs. In the case of our patient after RCM evaluation was performed it was recommended to the patient to remove the melanoma (lesion 1) and to follow-up the melanocytic nevus (lesion 2) with annual sequential digital dermoscopy (videodermoscopy) evaluation. Due to the patient’s request and concern both lesions were removed and sent for histopathology evaluation where lesion 1 was confirmed to be a melanoma (0.62 mm depth) and lesion 2 a dysplastic nevus. In conclusion, this case is a classic example where implementation of non-invasive screening methods can help confirm tumor diagnosis immediately at the bedside and help to reduce the waiting time for necessary removal of a melanoma and potentially reduce the unnecessary excision of a dysplastic nevus.

## Consent

Written informed consent for publication of patient clinical details and/or clinical/digital dermoscopy/RCM images was obtained from the patient.
