# Supplementary material for: Case Report: melanoma and melanocytic nevus differentiation with reflectance confocal microscopy
Source: F1000Res. 2015 Jul 15;4:257. [Version 1] doi: 10.12688/f1000research.6793.1 (PMC4513693; doi:10.12688/f1000research.6793.1)
Supplement: Peer review history

# Peer review: Referee response for version 1

**DOI:** 10.5256/f1000research.7299.r9603

**Published:** 2015-07-23

**Authors:** Joyce Lee, Sai Yee Chuah

**Affiliations:** 1 National Skin Centre, Singapore, Singapore; 2 Department of General Dermatology, National Skin Centre, Singapore, Singapore

This is a nice concise case report to increase our awareness of the availability of the new non-invasive tool (RCM), which can aid in early diagnosis of melanoma and hence, reducing the number of benign lesions excision especially for patients with atypical melanocytic nevi syndrome.

The knowledge added is limited but useful for other practitioners.

The title, abstract, article content and overall discussions are appropriate with good confocal and dermoscopy images attached. However, it would be nice to have histological images for correlation as well. The conclusions are sensible and balanced as well. However, it is important to indicate in the case report that the RCM is a useful complementary tool but should not replace clinical judgement on the necessity to closely monitor nevi and still offer excision if in doubt.

We have read this submission. We believe that we have an appropriate level of expertise to confirm that it is of an acceptable scientific standard.

# Peer review: Referee response for version 1

**DOI:** 10.5256/f1000research.7299.r9591

**Published:** 2015-07-22

**Authors:** Marco Ardigò

**Affiliations:** 1 Dermatology, San Gallicano Dermatological Institute, Rome, Italy

This is a well described case report on the use of RCM for melanoma and dysplastic nevi evaluation. Clinic, dermoscopic and confocal figures are well presented and clearly described the case.

I have read this submission. I believe that I have an appropriate level of expertise to confirm that it is of an acceptable scientific standard.
